# Hole Morphology and Keyhole Evolution during Single Pulse Laser Drilling on Polyether-Ether-Ketone (PEEK)

**DOI:** 10.3390/ma15072457

**Published:** 2022-03-26

**Authors:** Yanmei Zhang, Gang Yu, Chongxin Tian, Zhiyong Li, Jiayun Shao, Shaoxia Li, Xiuli He

**Affiliations:** 1 Institute of Mechanics, Chinese Academy of Sciences, Beijing 100190, China; zhangyanmei2@imech.ac.cn (Y.Z.); gyu@imech.ac.cn (G.Y.); tianchongxin@imech.ac.cn (C.T.); lizhiyong@imech.ac.cn (Z.L.); shaojiayun@imech.ac.cn (J.S.); 2 Center of Materials Science and Optoelectronics Engineering, University of Chinese Academy of Sciences, Beijing 100049, China; 3 School of Engineering Science, University of Chinese Academy of Sciences, Beijing 100049, China

**Keywords:** laser drilling, polyether-ether-ketone (PEEK), hole morphology, keyhole evolution, material-removal mechanism

## Abstract

Polyether-ether-ketone (PEEK), with its superior mechanical, chemical, and thermal properties, as well as high biocompatibility, has been used in aerospace, electronics, and biomedical applications. In this paper, a large number of experiments of single-pulse laser drilling on PEEK were performed to analyze the hole morphology and keyhole evolution, which were characterized by an optical microscope, charge-coupled device (CCD), and high-speed camera. A novel method is proposed to observe and measure the dimension of the processed hole rapidly right after laser drilling for special polymer materials with wear-resistance and non-conductivity. Morphological characteristics of holes are presented to illustrate the effect of pulse width and peak power on hole depth, hole diameter, and aspect-ratio. The obtained maximum drilling depth was 7.06 mm, and the maximum aspect-ratio was 23. In situ observations of the dynamic process of laser drilling, including the keyhole evolution together with ejection and vaporization behavior, were also carried out. The keyhole evolution process can be divided into three stages: rapid increment stage (0–2 ms) at a rate of 2.1 m/s, slow increment stage (2–4 ms) at a rate of 0.3 m/s, and stable stage (>4 ms). Moreover, the variation of dimensionless laser power density with the increase in pulse width was calculated. The calculated maximum drilling depth based on energy balance was compared with the experimental depth. It is proven that the laser–PEEK interaction is mainly influenced by a photothermal effect. Ejection is the dominant material-removal mechanism and contributes to over 60% of the depth increment during the rapid increment stage, while vaporization is dominant and contributes to about 80% of the depth increment during the slow increment stage. The results reveal the material removal mechanism for single-pulse laser drilling on PEEK, which is helpful to understand the dynamic process of keyhole evolution. This not only provides a processing window for future laser drilling of PEEK but also gives a guide for the manufacturing of other polymers.

## 1. Introduction

Polyether-ether-ketone (PEEK) is an aromatic thermoplastic polymer with semi-crystalline structure. It is used in a variety of areas, especially in the aerospace, automotive, and electronics industries, due to its superior mechanical, chemical, and thermal properties [1,2]. Moreover, because of its high biocompatibility and high temperature resistance, PEEK is widely used in the medical industry for dental materials and bone repair [3,4,5]. For many weight-critical components in the aerospace industry such as the new A350 and the B373 aircrafts, PEEK and its polymer materials have become the major structural materials to substitute metals when manufacturing fuel filters, bolts, nuts, bobbins, etc. [6]. The drilling technique is very important in the machining process, as millions of holes are needed to produce riveted and bolted joints for the assembly operation [7,8]. In biomedical applications, a PEEK matrix with high biocompatibility is often processed into microscale cavities or holes. The distribution and size of the holes play an important role in osteogenic differentiation and surface texture, which affect the wettability of the material [2,9,10,11].

On the drilling of PEEK or other polymer materials, conventional machining techniques, such as twist drilling and milling, are usually associated with delamination damage, poor surface quality, low dimensional accuracy, and severe tool wear [6,12,13], as the drilling tool, twist drill or polycrystalline diamond (PCD), usually undergoes high thrust forces and severe tool wear. The frequent replacement of cutting tools incurs extra production time and cost. Moreover, it is hard to get holes with a small diameter (<0.5 mm) and high aspect-ratio due to the limitation of tool size [14,15]. Unconventional methods such as abrasive water jet drilling (AWJ), ultrasonic machining (USM), and laser beam machining (LBM), have also been used recently. AWJ has the limitations of high operation cost, abrasive slurry collection, and low material removal rate, despite producing holes with small thermal damage [16,17]. A low material removal rate and tool wear also limit the utilization of USM [18]. As a promising alternative method, laser processing has the characteristics of a small spot radius, high power density, and strong directivity [19,20,21]. It is easy to realize the high precision and automation of drilling. Compared with other processing technologies, it imparts many advantages including the processing of micro-holes and thin kerf width, no toolwear or frequent replacement of tools, higher drilling speed, and absence of drilling force, abrasives, and liquid media [6,16,22]. With these advantages, laser drilling has increasingly been used in the processing of PEEK and other polymers.

For the machining of micro-holes, the hole morphology is a core feature, which determines the quality and performance of the hole. There have been some experimental studies on the hole morphology for laser drilling of PEEK and polymer materials. Micro-holes on a number of polymers (polyethylene terephthalate (PET), polyimide (PI), polycarbonate (PC), polystyrene (PS), polymethyl methacrylate (PMMA), and PEEK) were obtained using an ultraviolet excimer KrF laser with multi-pulse radiation [23]. The hole diameter was in the range of 10 μm to 100 μm, and the hole depth was up to 20 mm after drilling 7500 pulses. A pulse laser of Nd:YAG at the frequency of 10 Hz and pulse width of 10 ns was used to produce the microstructure on a PEEK sample with a thickness of 1 mm [11]. Micro-holes with 20 μm diameter enhanced the wetting properties of the PEEK surface. Laser micro-structuring of PEEK was demonstrated with femtosecond laser pulses [24]. The difference in ablation threshold and morphology of the ablation spot was found using a single-pulse laser with wavelengths of 775 nm and 387 nm. Although some progress has been made on the study of hole morphology for laser drilling on PEEK and other polymers, the relationship between processing parameters and hole morphology, i.e., the parameter optimization for target hole shape, such as a high aspect ratio, remains to be further studied. In addition, sampling for observation and measurement of micro-hole morphology is very challenging.

For the observation of the dimension and morphology of micro-holes after experiments, it is necessary to get a central section of the processed hole, which is very difficult due to the small diameter in the range of 10 μm to 100 μm. A Leica digital microscope was used to measure the depth of the hole (<50 μm) introduced by the irradiating laser on a PEEK and carbon fiber lamina [22]. Laser scanning confocal microscopy (LSCM) is usually used for shallow holes or microgrooves [25]. Temporal variations of the secondary speckle on the drilled glass samples have been used to estimate the depth, with a laser, ultrasound transmitter, and camera [26]. Furthermore, a 3D optical profiling system and thermal field-emission environment scanning electron microscope were utilized to observe the morphology of laser-drilled microhole array electrodes [27]. In the case of deep holes, destructive measurement methods were employed, such as wire cutting, grinding, and polishing. SS304 or Ni alloy was split using sandpapers and polishing agents [28]. Then, they were cleaned to wash off the fragments caused by the sandpapers or polishing agents to measure depth. Grinding and polishing samples using various grades of SiC paper were also used in single-pulse laser drilling of multi-layer carbon fiber composites [29]. With all these methods, it is hard to accurately find the central section. Consequently, the morphologic characteristics of the hole are not fully observed. Moreover, it takes a lot of time to deal with these samples, especially for multi-group experiments, which adds extra time and cost.

The dynamic evolution during the drilling process is also very important in determining the final hole morphology. In order to observe and analyze the physical processes of the laser drilling, an in-situ observation system was used in [30]. The plasma morphology, evolution, melt ejection, and expulsion could be analyzed, which were hard to obtain after laser drilling. In the femtosecond laser drilling of PMMA, an imaging system and illuminator with lens and CCD were applied on the side view of PMMA, which were perpendicular to the direction of the laser beam [31]. The dynamics of the hole formation and ablation plume physics were examined in time-resolved side-view images recorded with an intensified-CCD camera during the laser drilling process of glasses [32]. The transient effect and ablation plume confirmed the build-up of heat accumulation effects during the pulse train. Nevertheless, the above observation only focused on the evolution of hole depth. Physical processes and transient phenomena during laser drilling of polymers in time-resolved observations have not been examined and analyzed. The material removal mechanism cannot be fully explored without a fundamental understanding of the keyhole evolution.

Therefore, in order to investigate the effect of peak power and pulse width on the hole morphology and dynamic evolution of keyhole during single-pulse laser drilling on PEEK, a series of fundamental experiments of laser drilling were conducted. A new method is proposed, which provides a simple and effective way to rapidly observe and measure the depth and diameter of the processed hole right after drilling experiments, especially in the field of laser drilling on polymer materials with non-conductivity, non-transparency, and wear-resistance. Furthermore, the dynamic evolution process during laser drilling was captured by in-situ optical observation. Dimensionless laser power density was defined and used to analyze the driving mechanism of laser drilling. The calculated maximum drilling depth based on energy balance was compared with experimental depth. The results discussed in this study not only provide a processing window for laser drilling of PEEK and other polymers, but also reveal the dynamic process of laser drilling to better understand the material removal mechanism of the laser–PEEK interaction.

## 2. Experimental Method and Material

**Figure 1:**
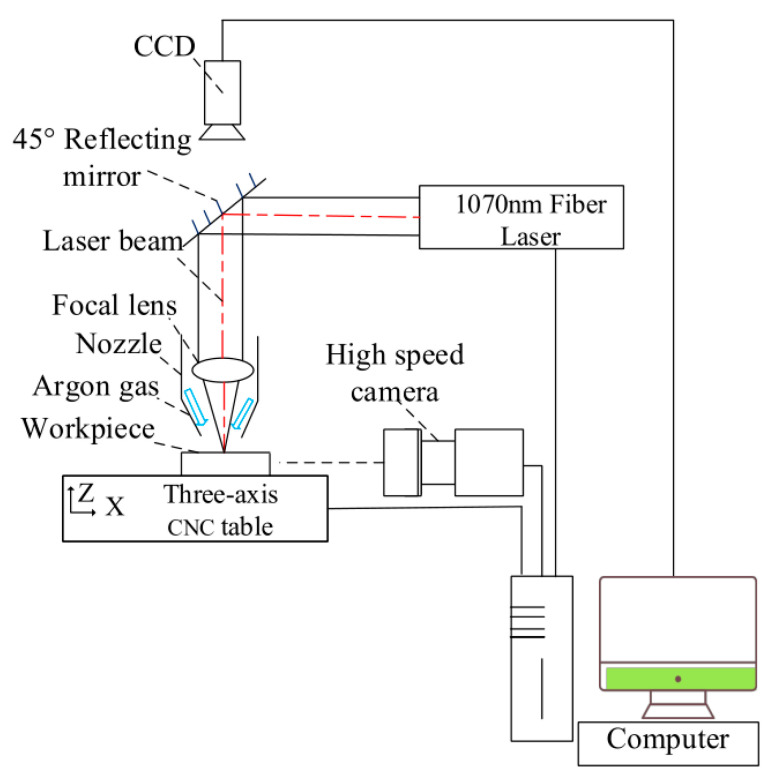
Schematic diagram of the experimental setup of laser drilling.

All experiments were conducted on an infrared laser micro-processing system. The device consisted of a 1070 nm ytterbium fiber laser with continuous wave (YLR-1000-WC, IPG, Oxford, USA), optical system, fiber laser cutting head (LC209, GAUTEK, Beijing, China), three-dimensional computer numerical control (CNC) system, high-speed camera observation system (I-SPEED 221, ix-cameras, Rochford, UK), and charge-coupled device (CCD) image system. A schematic diagram of the experimental setup is shown in Figure 1.

Laser output can be modulated as a series of pulse trains up to 50 kHz. The output laser beam is guided via a set of mirror reflectors to the fiber laser cutting head, equipped with a focal lens and a coaxial gas nozzle for drilling. The waist diameter and the Rayleigh range of the laser beam focused by a lens of 150 mm focal length are approximately 21 μm and 300 μm, respectively. The nominal output power is up to 1000 W, and the shortest pulse duration is as low as 10 μs. The beam quality M^2^ is 1.05, close to TEM_00_ mode. A standard rectangular pulse mode was used in this work. The single pulse energy is determined by both the peak power and the pulse width. The peak power *P_peak_* refers to the maximum amount of power delivered in a pulse duration. It is defined as the ratio of the energy to the time. The peak power can be regarded as constant in one pulse train as the laser is quasi-continuous, and it is given by Equation (1). The laser power density per pulse at the focal plane is between 3 MW/cm^2^ and 72 MW/cm^2^. The peak power and pulse width were varied in this study. Their impact on hole morphology and keyhole evolution was further investigated.

(1) $$E=P_{peak}\tau_{L},$$

where *E* is the single pulse energy, *P_peak_* is the peak power, and $\tau_{L}$ is the pulse width.

**Figure 2:**
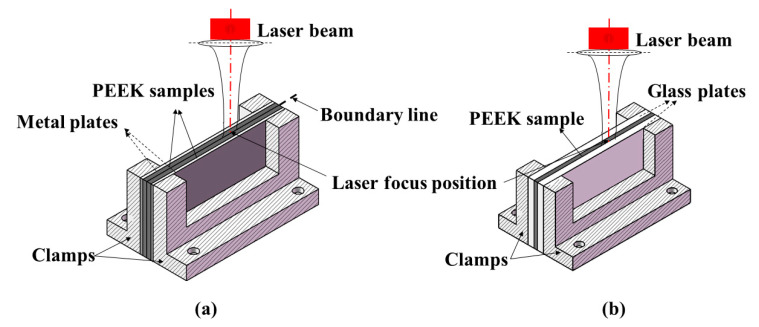
Schematic diagram for sample clamping device with sandwich structure: (**a**) two identical PEEK samples pressed together by two metal plates; (**b**) one PEEK sample sandwiched between two glass plates.

In this paper, a novel high-efficiency laser drilling method is proposed to observe and investigate the morphologic characteristics of micro-holes rapidly right after laser drilling experiments. PEEK samples were mounted on the self-developed clamps with a “sandwich” structure, as shown in Figure 2a. The thicknesses of the PEEK and metal plate were 3 mm and 2.5 mm, respectively. In order to achieve accurate positioning of the hole, two identical samples were pressed together by two metal plates. PEEK samples and metal plates were tightly fixed in place by clamps. The laser spot was precisely focused on the boundary line of the two samples with the aid of a charge-coupled device (CCD) and Dino-lite digital microscope. The sectional morphology of the hole can be obtained when separating two sheets after drilling, and then the dimension and morphology of the hole can be examined. The new drilling method provides an accurate and time-saving approach to observe the hole morphology.

In order to observe dynamic evolution process of the keyhole during laser drilling, a high-speed camera and glass plates were used, as shown in Figure 2b. One PEEK sample was sandwiched between two clamps. Two glass plates were used as transparent walls which provided constraints to simulate the actual drilling process. The laser spot was precisely focused on the boundary line between PEEK and the glass plate with the aid of a CCD and Dino-lite digital microscope. All clamps were fixed on a three-axis CNC table. For recording the whole process of keyhole evolution, a high-speed camera was applied on the side view of the PEEK sample perpendicular to the direction of the laser beam. Furthermore, the position of the camera was between the laser head and the upper surface of PEEK for observation of the dynamic process at the entrance of the hole. The resolution, recording rate, and shutter speed were set from 144 × 230 pixels to 128 × 40 pixels, 20,000 fps to 100,000 fps, and 1/500,000 s to 1/100,000 s, respectively. The recording rate and shutter speed were also optimized specifically to avoid image overexposure. Under this condition, the dynamic evolution process of the keyhole could be clearly recorded.

PEEK5600G was used as experimental material in this work. The dimensions of the specimen were 100 mm × 50 mm × 3 mm. Major thermal and physical properties are listed in Table 1. Before conducting experiments, the samples were ground and polished using various grades of SiC paper (400#, 800#, 1000#, and 2000#) in turn. Then, the samples were cleaned in anhydrous alcohol with an ultrasonic treatment for 5 min.

Holes were drilled on the focal plane by a single pulse laser; hence, the depth of focus of the laser beam after focusing on the sample was 0 mm. Argon was employed at a pressure of 0.4 MPa, and the high-pressure assisted gas jetted out from the nozzle with a 2 mm distance away from the surface of workpiece. The processing parameters studied in this paper were changed in the following range: laser peak power from 100 W to 1000 W and pulse width from 50 μs to 20 ms. In order to ensure the reliability and stability of the results, three holes were drilled for each processing parameter set. The interval of holes was set as 8 mm to avoid the spatter influence on the adjacent holes. After conducting experiments, two samples were separated. A Dino-lite digital microscope AM4115ZT and CCD were used to observe the cross-section of processed holes. An MD-50 UM200i optical microscope was used for observation and measurement of morphologic characteristics, which mainly involved depth and diameter of the hole.

## 3. Results and Discussion

### 3.1. Hole Morphology

The dimension and morphology of micro-holes are important for the performance of any machined parts [7]. The diameter and depth of the hole are regarded as important indices to evaluate the geometry and material-removal efficiency [34], respectively. The aspect-ratio is defined as the ratio of hole depth to hole diameter [35]. Micro-holes with small diameter, high-aspect ratio, and required precision are needed for machined structures in industrial applications [31,36]. Therefore, the hole depth, hole diameter, and aspect ratio were investigated in this paper to evaluate the morphology for single-pulse laser drilling on PEEK.

#### 3.1.1. Effect of Laser Power

Using the self-developed experimental device, shown in Figure 2a, the morphology of the half-hole on separate PEEK sheets was observed, and the dimension was measured three times to reduce the uncertainty. The average mean of six sets of data was calculated, taken as the final diameter, depth, and aspect-ratio. The influences of peak power and pulse width on the hole diameter, hole depth, and aspect-ratio were elucidated.

**Figure 3:**
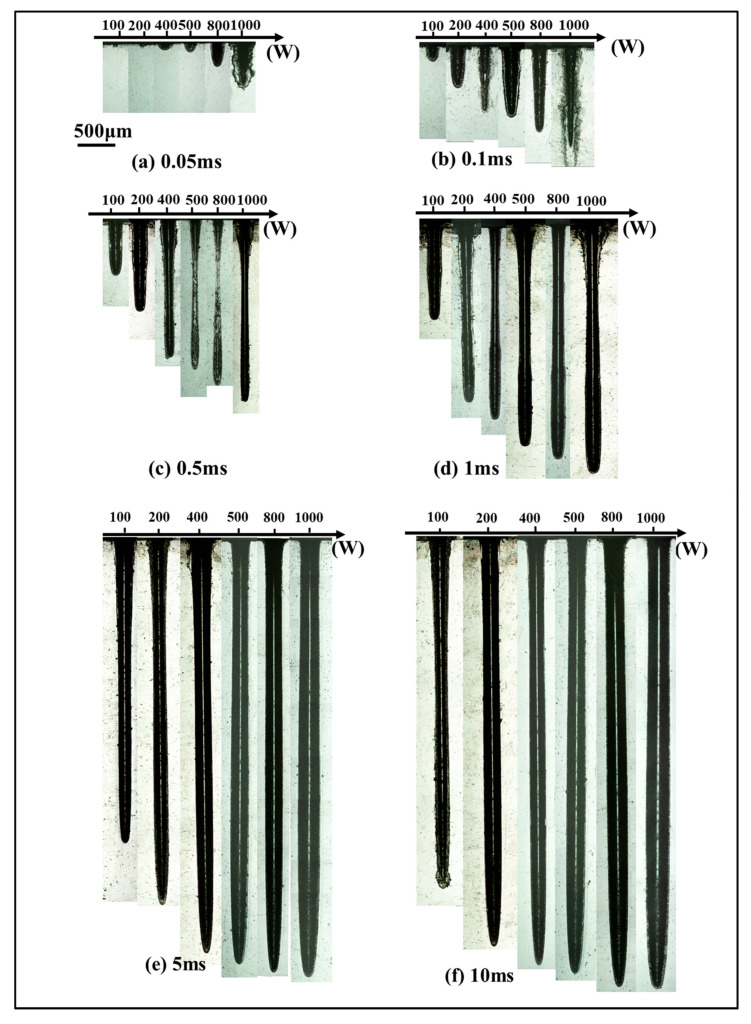
Sectional morphology of the hole under different peak powers with pulse widths of (**a**) 0.05 ms, (**b**) 0.1 ms, (**c**) 0.5 ms, (**d**) 1 ms, (**e**) 5 ms, and (**f**) 10 ms.

Figure 3 illustrates the sectional morphology of the hole with the increase in peak power when the pulse width was constant. For a pulse width of 0.05 ms, hole depth was less than 1 mm although peak power increased from 100 W to 1000 W. When the pulse width was 0.1 ms and the peak power was 800 W, the hole depth reached 1 mm. A deeper hole was obtained with a higher peak power and longer pulse width. It can be noticed that, when the pulse width was less than 5 ms, a chamfer was formed at the entrance edge of the hole with the increase in peak power. Due to the increase in peak power and pulse width, more thermal erosion occurred on the entrance surface of the hole, which caused an increase in the entrance angle. To a certain extent, with the pulse width further increasing, the chamfer was not apparent. The outer edges of the keyhole were parallel when the pulse width was up to 5 ms.

**Figure 4:**
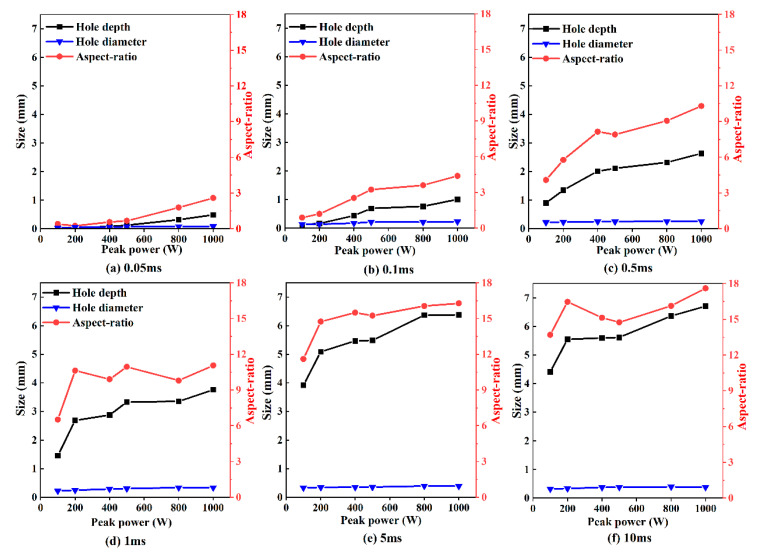
Hole depth, hole diameter, and aspect ratio under different peak powers with pulse widths of (**a**) 0.05 ms, (**b**) 0.1 ms, (**c**) 0.5 ms, (**d**) 1 ms, (**e**) 5 ms, and (**f**) 10 ms.

The curves in Figure 4 show the variation of the hole depth, hole diameter, and aspect ratio with the increase in peak power, when the pulse width was at 0.05 ms, 0.1 ms, 0.5 ms, 1 ms, 5 ms, and 10 ms. It can be noted that, when pulse width was less than 5 ms, a higher peak power led to a deeper drilled hole and larger hole diameter. When the pulse width was varied from 0.05 ms to 0.5 ms, the aspect-ratio increased rapidly with the increase in peak power, but it was less than 10. In order to obtain a larger aspect-ratio (>10), it is necessary for the pulse width to exceed 1 ms. At the pulse widths of 5 ms and 10 ms, the differences in hole depth, hole diameter, and aspect-ratio were not significant.

#### 3.1.2. Effect of Pulse Width

**Figure 5:**
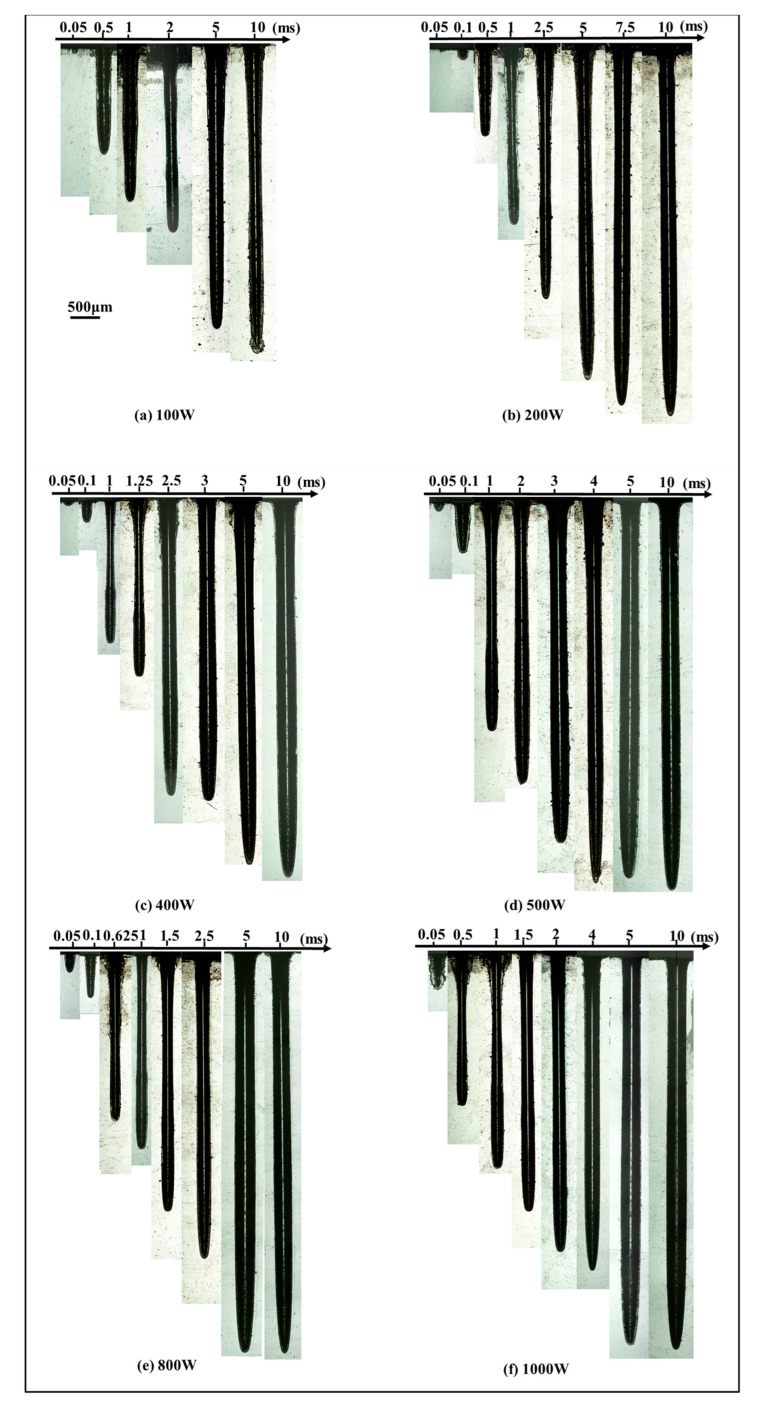
Sectional morphology of the hole under different pulse widths with the peak power of (**a**) 100 W, (**b**) 200 W, (**c**) 400 W, (**d**) 500 W, (**e**) 800 W, and (**f**) 1000 W.

**Figure 6:**
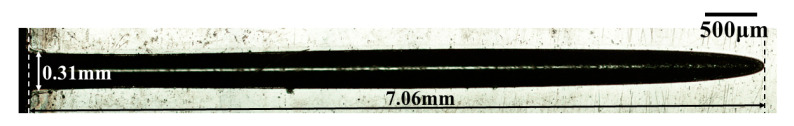
Sectional morphology of the hole with the maximum aspect ratio.

Figure 5 illustrates the sectional morphology of the hole with the increase in pulse width when the peak power was constant. A hole could not be formed when the pulse width was less than 0.05 ms at the peak power of 100 W, 200 W, and 400 W, respectively. With the peak power varying from 100 W to 1000 W, the hole depth increased rapidly in the range of pulse width from 0.05 ms to 2 ms. A slow increment in the hole depth followed from 2 ms to 4 ms. When the pulse width exceeded 4 ms, the hole depth was almost unchanged. Under the laser parameters used, the obtained maximum aspect-ratio of the hole was 23. The sectional morphology of the hole with the maximum aspect-ratio is enlarged in Figure 6. It is shown that the hole depth was 7.06 mm and the hole diameter was 0.31 mm.

**Figure 7:**
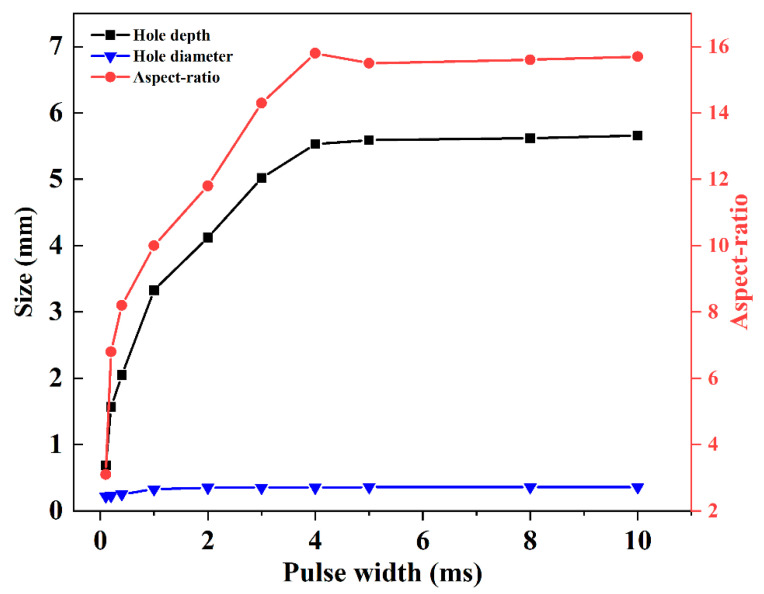
Hole depth, hole diameter, and aspect ratio under different pulse widths at the peak power of 500 W for single-pulse laser drilling.

When the peak power was at 500 W, the increases in hole depth, hole diameter, and aspect ratio with the variation of pulse width are shown in Figure 7. It is shown that the hole diameter increased with the increase in pulse width in the first 2 ms. After 2 ms, the hole diameter hardly increased. The hole depth increased rapidly with the increase in pulse width during the first 2 ms. Fast and deep ablation at an approximate rate of 2.1 m/s was observed. When the pulse width was from 2 ms to 4 ms, slow ablation at an increment rate of 0.3 m/s was found, with a nearly one-seventh increment rate of that for the first 2 ms. It can be noted that a longer pulse width led to a larger aspect-ratio during the first 4 ms. This is because the hole depth increased more rapidly compared with the hole diameter. When the pulse width exceeded 4 ms, the hole diameter and hole depth were almost unchanged with the increase in pulse width. Moreover, there was no significant difference in the aspect-ratio with the pulse width varying from 4 ms to 10 ms.

On the basis of the above analysis, the increments in hole depth, hole diameter, and aspect-ratio depended on the increase in pulse width and peak power. The energy accumulation increased with the increase in effective laser power and interaction time between the laser and PEEK. By comparing Figure 4 with Figure 7, it can be noted that the hole diameter was more dependent on pulse width than peak power. Additionally, the increment in hole depth with the increase in pulse width was larger than that with the peak power, which means that pulse width had a more important effect on hole morphology than peak power. The hole depth, hole diameter, and aspect-ratio first increased rapidly with the increase in pulse width for the first 2 ms, and then increased slowly. Once the pulse width reached 4 ms, it did not play an important role in the increments in hole depth, hole diameter, and aspect-ratio.

### 3.2. In Situ Observation of Laser Drilling Process

On the basis of the geometric features of the above-obtained holes, the effects of peak power and pulse width on the hole depth, hole diameter, and aspect-ratio were summarized for single-pulse laser drilling. However, these results were only obtained after laser drilling, which was an after-event analysis, not an online investigation. This is not enough to adequately understand the transient evolution of the keyhole and material-removal mechanism of the laser–PEEK interaction. Therefore, in order to better observe the physical process of laser drilling, an in situ observation of laser drilling on PEEK was conducted.

#### 3.2.1. Keyhole Evolution

The evolution of the keyhole was captured by a high-speed camera. The dynamic processes of the keyhole with different pulse widths (0.8 ms, 1 ms, and 5 ms) were recorded when the peak power was 500 W. A series of pictures were obtained. At the pulse widths of 0.8 ms and 1 ms, the keyhole evolution showed a similar tendency to that at the pulse width of 5 ms. Next, a pulse width of 5 ms was chosen to analyze the keyhole evolution.

**Figure 8:**
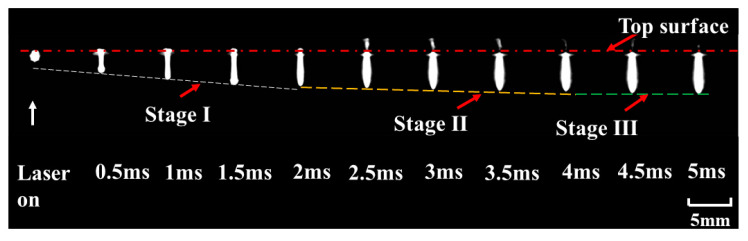
Keyhole evolution with the pulse width of 5 ms during single-pulse laser drilling at a peak power of 500 W.

**Figure 9:**
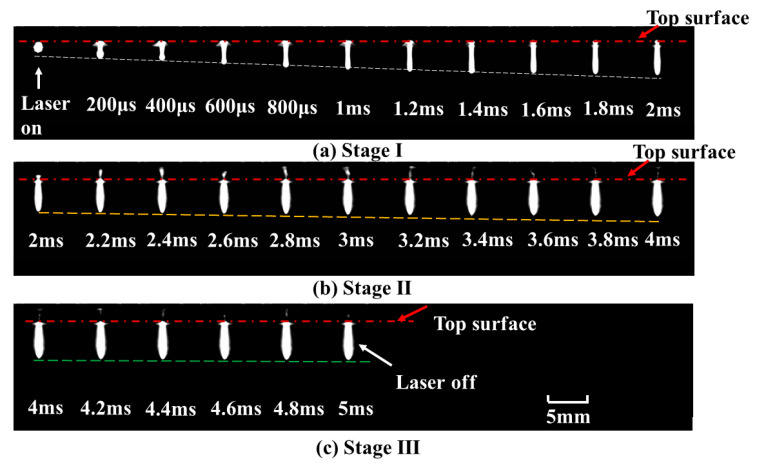
Three stages of keyhole evolution with a pulse width of 5 ms during single-pulse laser drilling at a peak power of 500 W. (**a**) stage I: 0–2 ms, (**b**) stage II: 2 ms–4 ms, (**c**) stage III: 4 ms–5 ms.

The optimal resolution, frame rate, and shutter speed were 144 × 230 pixels, 21,102 fps, and 2 μs, respectively. The frames with the interval of 0.5 ms are shown in Figure 8. It can be noted that the depth and diameter of the keyhole evolved with time until the laser was turned off. According to the drilling rate, keyhole evolution can be divided into three stages. The keyhole depth underwent a process from rapid increment to slow increment, before hardly increasing. The three stages of keyhole evolution are shown in Figure 9.

It can be obviously observed that there were two different increment rates of the depth with an increase in pulse width, as shown in Figure 9a,b. Three stages were clearly indicated: a rapid increment stage at an approximate rate of 2.1 m/s, a slow increment stage at a rate of 0.3 m/s, and a stable stage. For stage I, the depth of the keyhole increased rapidly with the increase in pulse width during the first 2 ms. For stage II, the depth of the keyhole increased slowly from 2 ms to 4 ms, implying a lower material removal rate after 2 ms. For stage III, when the pulse width exceeded 4 ms, a stable hole was formed. The depth of the keyhole remained almost constant, as shown in Figure 9c. The measured hole size after laser drilling was consistent with the in-situ observation of keyhole evolution.

#### 3.2.2. Vaporization and Ejection

During laser drilling, when the laser is irradiated on the surface of the material, the irradiated area absorbs the laser energy, and available energy is instantaneously converted into heat. The high laser power density results in an instantaneous temperature rise on the surface of the material, which exceeds the melting and vaporization temperature, leading to thermal expansion, melting, and vaporization of the material. The molten material is ejected from the matrix by the vaporization-induced recoil pressure [37,38]. As the keyhole evolves, the mass of the material is lost by ejection and vaporization.

**Figure 10:**
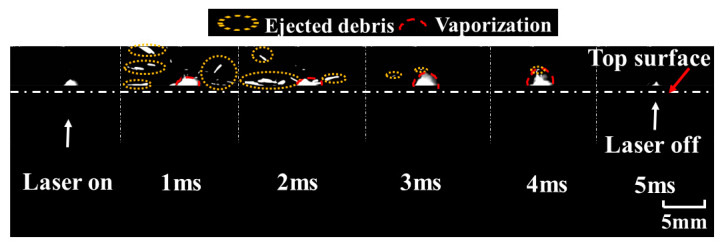
Ejection and vaporization behavior with a pulse width of 5 ms during single-pulse laser drilling at a peak power of 500 W.

**Figure 11:**
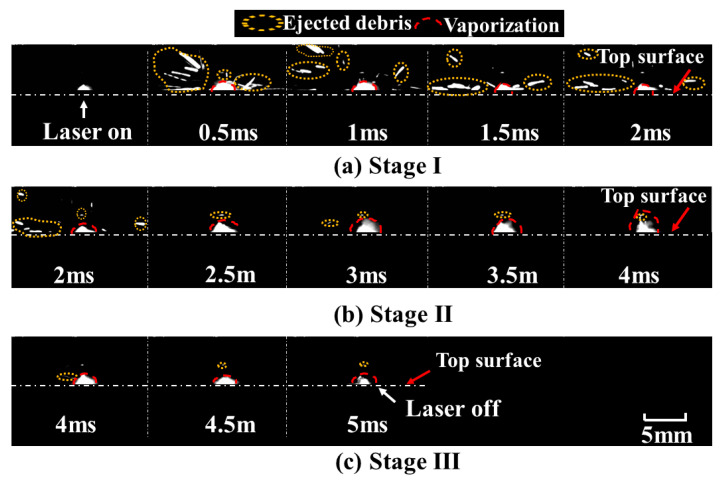
Ejection and vaporization behavior for three stages with a pulse width of 5 ms during single-pulse laser drilling at a peak power of 500 W. (**a**) stage I: 0–2 ms, (**b**) stage II: 2 ms–4 ms, (**c**) stage III: 4 ms–5 ms.

In order to better understand the material-removal process during keyhole evolution, the dynamic process at entrance of the hole with a pulse width of 5 ms was also observed. A series of images were captured, as shown in Figure 10. The ejection and vaporization behavior were closely related to the keyhole evolution during the whole process of laser drilling. When the laser was on, there was a highlighted region around the entrance of the keyhole in the initial 100 μs. Followed by a reduction in the bright area, ejected debris with different shapes was clearly observed above the entrance of the keyhole after 100 μs. Moreover, vaporization was found at this stage. During the first 2 ms, there was intense ejection and less vaporization. After 2 ms, a small amount of ejection was observed, but the evaporation persisted until the laser was turned off. The dynamic processes for different stages are shown in Figure 11.

At the rapid increment stage, there was a highlighted region within the initial 100 μs. This stage was accompanied by intense ejected debris and vaporization at the entrance of the hole; therefore, the depth of keyhole increased rapidly during the first 2 ms. Due to the upward and circumferential ejection and vaporized material at higher power density, a chamfer was formed at the entrance edge of the hole, as shown in Figure 3a–d. At the slow increment stage, as the depth increased with the increase in pulse width, it was dominated by evaporation after 2 ms until the hole depth hardly increased. With the ejection decreasing, the chamfer angle became smaller, as shown in Figure 3e–f. At the stable stage, the keyhole began to bulge. This might have been caused by multiple reflections on the keyhole wall and the erosive effect at high temperature. Consequently, the depth increased more rapidly during the first 2 ms than during the remaining 3 ms, implying a lower material removal rate after 2 ms.

### 3.3. Material Removal Mechanism

Photothermal and photochemical effects are the two main mechanisms contributing to material removal [39,40,41]. For a laser with ultraviolet wavelength and ultrashort duration (picosecond or femtosecond), photochemical excitation is regarded as the main factor directly impacting the bond scission. For a laser with infrared wavelength and long duration (millisecond or microsecond), the photothermal effect is dominant. The laser energy is subsequently converted into heat, contributing to material removal [42,43,44,45]. In this work, in the case of the used laser wavelength (1070 nm) and pulse width (millisecond and microsecond), the photothermal effect was regarded as the main contributor to the material removal.

**Figure 12:**
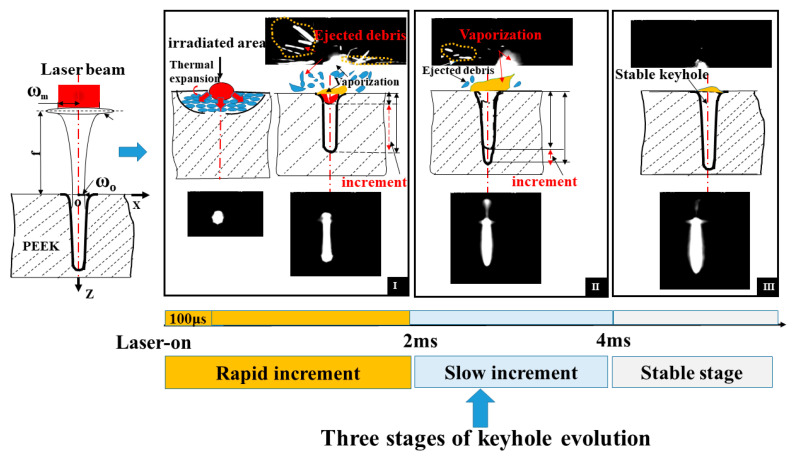
Material removal mechanism during the single-pulse laser drilling of PEEK.

According to the in-situ observation of vaporization and ejection in Section 3.2.2, the material removal mechanism for single-pulse laser drilling on PEEK is proposed in Figure 12. Vaporization or ejection mainly contributes to the material removal at the three stages of keyhole evolution: rapid increment stage, slow increment stage, and stable stage. During the rapid increment stage in the first 2 ms, there is a temperature gradient along the depth direction of material because of the thermal expansion in the initial 100 μs. After 100 μs, there is intense ejected debris at the entrance of the hole, accompanied by thermal vapor. The keyhole evolution is dominated by ejected debris at this stage. During the slow increment stage from 2 ms to 4 ms, as the amount of ejection decreases, there is a lot of vaporization at the entrance of the hole. Evaporation is the main material removal process at this stage. At the stable stage after 4 ms, there is little vaporization at entrance of the hole, such that the hole depth no longer increases.

#### 3.3.1. Dimensionless Laser Power Density

The vaporization or ejection behavior differs with the increase in pulse width, which is closely related to the change in laser power density. It is important to note that ejection is possible only when the laser power density exceeds the threshold density. In order to understand the transition of dynamic process from ejection to vaporization, the dimensionless laser power density can be used to analyze the driving mechanism in laser drilling [37]. In this paper, the distribution of laser beam is assumed to be Gaussian, as shown in Equation (2).

(2) $$I(r)=I_{0}\exp\left(-\frac{2r^{2}}{w^{2}}\right),$$

where *I*_0_ is the maximum laser power density at the beam center, *r* is the radius from the beam center, and *w* is the radius of the beam where the beam intensity falls to e^−2^. The maximum laser power density is given by Equation (3).

(3) $$I_{0}=\frac{2aP}{\pi w^{2}},$$

where *a* is the absorption coefficient of PEEK, and *P* is the peak power. For a Gaussian beam propagating in space, the beam radius *w*, which is at a vertical distance *z* from the focus position, can be calculated by Equation (4).

(4) $$w\left(z\right)=w_{0}\cdot \sqrt{1+{\left(\frac{z}{z_{0}}\right)}^{2}},$$

where *w*_0_ is the beam radius at the focus point, *z* is the vertical distance from the focus plane, and *z*_0_ is the Rayleigh range. The dimensionless laser power density is defined by Equation (5) [37].

(5) $$I^{*}=\frac{w\left(z\right)\cdot I_{0}\cdot C_{pl}}{h_{lv}\cdot k},$$

where *C_pl_*, *h_lv_*, and *k* are the specific heat, latent heat of vaporization, and thermal conductivity of PEEK, respectively. The specific heat and latent heat of vaporization with temperature can be calculated by Equations (6) and (7) [33,46].

(6) $$C_{pl}=0.496T+308.15,$$

(7) $$h_{lv}=\int_{T1}^{T2}C_{pl}dT,$$

where *T*, *T*_1_, and *T*_2_ denote the temperature, liquid temperature, and gaseous temperature.

**Figure 13:**
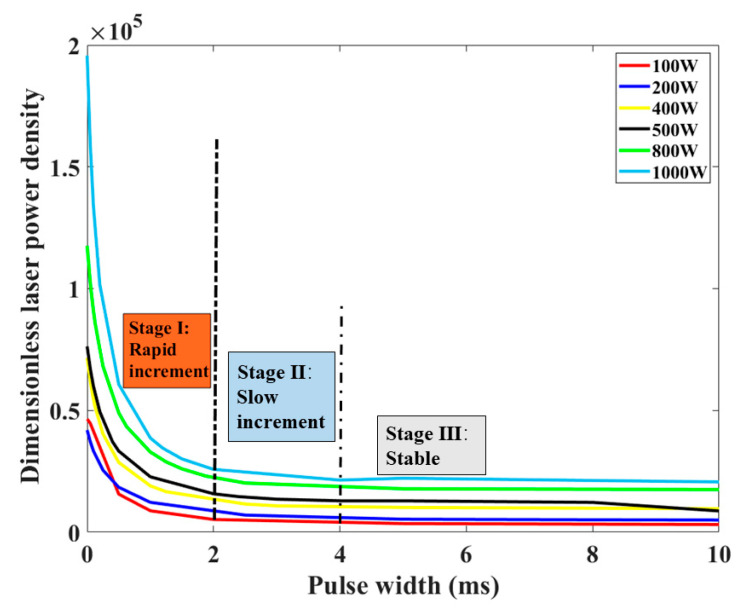
Variation of dimensionless laser power density with pulse width under different peak power.

In order to investigate the relationship between dimensionless laser power density and material removal mechanism, Equation (5) can be further expressed as Equations (8) and (9).

(8) $$I^{*}=\frac{w^{2}\cdot I_{0}}{\rho \cdot w\cdot h_{lv}\cdot \alpha }=\frac{\mathrm{be}\;\mathrm{absorbed}\;\mathrm{power}}{\mathrm{vaporisation}\;\mathrm{energy}},$$

(9) $$\alpha =\frac{k}{\rho \cdot C_{pl}},$$

where *ρ* is the density of PEEK, and *α* represents the thermal diffusivity. It is constant for the given material. Therefore, the dimensionless power density I* defines the ratio of laser absorption energy to the vaporization energy used for material removal. Combining the processing parameters and related thermophysical parameters in Table 1, the variation of dimensionless laser power density with the increase in pulse width is shown in Figure 13.

For different peak power varying from 100 W to 1000 W, the dimensionless laser power density decreased with the increase in pulse width. It can be noted that the variation of dimensionless laser power density is closely related to the above-defined three stages of keyhole evolution. During the first 2 ms, the dimensionless laser power density is much higher. Hence, there is a rapid increase in keyhole depth at this stage. When the pulse width ranges from 2 ms to 4 ms, the dimensionless laser power density decreases sharply compared with the first 2 ms, causing a slow increase in the depth. When a stable hole is formed after 4 ms, the dimensionless laser power density changes little.

#### 3.3.2. Relative Importance of Vaporization and Ejection

**Figure 14:**
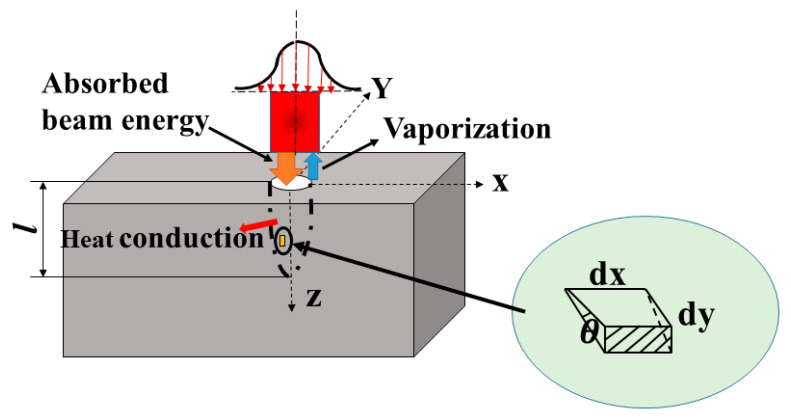
Control surface based on energy balance during single-pulse laser drilling process.

In order to verify the presence of ejected debris, which is the main material removal mechanism at the rapid increment stage, it is assumed that evaporation is the only mechanism present in the drilling process. Since vaporization is the only material removal mechanism, it is possible to use an infinitesimal control surface to analyze the machined zone [47]. The control surface during the laser drilling process is shown in Figure 14. On the basis of the energy balance, the energy for laser absorption, material evaporation, and heat conduction is expressed in Equation (10).

(10) $$E_{ab}\left(x,y\right)dxdy=E_{vap}\left(x,y\right)dxdy+E_{cond}\left(x,y\right)dA,$$

For the infinitesimal control surface at the point (*x*_0_, *y*_0_), *E_ab_*, *E_vap_*, and *E_cond_* denote the laser absorption energy, vaporization energy, and conduction energy, respectively. In Equations (2)–(4), considering the spatial distribution of the beam energy, *E_ab_* on a point (*x*_0_, *y*_0_) from the beam center, at a distance *x*_0_ along the *x*-axis and *y*_0_ along the *y*-axis from the beam axis, is given by Equation (11).

(11) $$E_{ab}\left(x_{0},y_{0}\right)=\int_{-\infty }^{x_{0}}\frac{2aP}{\pi w^{2}\cdot v}\exp\left(-\frac{2\left(x^{2}+y_{0}{}^{2}\right)}{w^{2}}\right)dx,$$

where *v* is the drilling speed. The vaporization energy associated with the depth is the energy used to heat the material to the vaporization temperature plus the vaporization heat capacity of the material. It is given by Equation (12).

(12) $$E_{vap}\left(x_{0},y_{0}\right)=\rho \cdot C_{pl}\left(T_{vap}-T_{0}\right)\cdot l\left(x_{0},y_{0}\right)+\rho \cdot h_{lv}\cdot l\left(x_{0},y_{0}\right),$$

where *T_vap_* is the vaporization temperature, *T*_0_ is the ambient temperature, and *l* (*x*_0_, *y*_0_) is the hole depth at the point (*x*_0_, *y*_0_). The heat transferred into the material through conduction can be calculated by Equation (13).

(13) $$E_{cond}=2\cdot k\cdot \sqrt{\frac{\tau_{L}}{\pi \cdot \alpha }}\left(T_{vap}-T_{0}\right),$$

where *τ_L_* is the interaction time between the laser beam and the material. From the geometry of the infinitesimal surface element in Figure 14, it is evident as shown in Equation (14).

**Figure 15:**
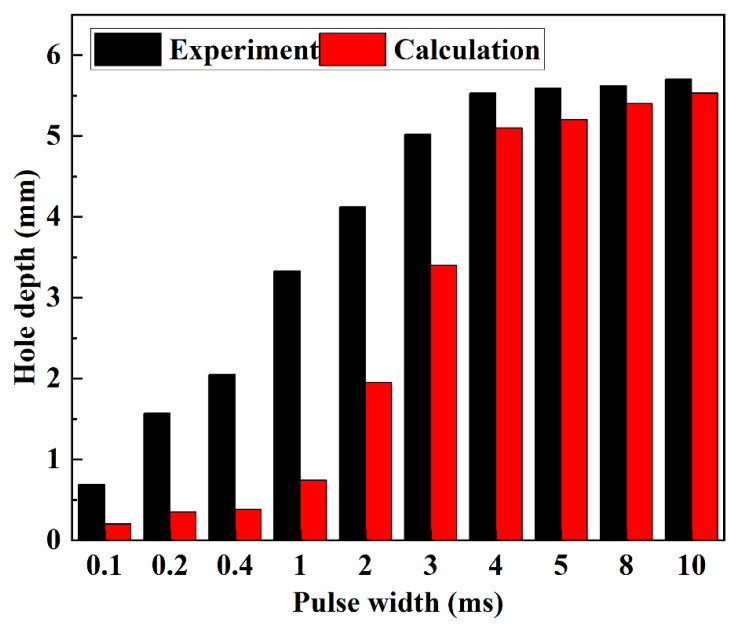
Experimental and calculated hole depth under different pulse width at the peak power of 500 W.

(14) dA=dxdy⋅tanθ,

where *θ* is the average slope of the hole, which is given by Equation (15).

(15) $$\theta =\tan^{-1}\left(\frac{h}{r_{top}-r_{bot}}\right),$$

where *r_top_* is the entrance diameter and *r_bot_* is the exit diameter. Because all processed holes are blind holes, *r_bot_* is equal to 0. Substituting Equations (11)–(15) into Equation (10), the maximum hole depth can be obtained at the beam centerline by putting *x*_0_ = ∞ and *y*_0_ = 0. This is given by Equation (16).

(16) $$l_{\max}=\frac{\frac{\sqrt{2}a\cdot P}{w\cdot v\cdot \sqrt{\pi }}-2k\cdot \sqrt{\frac{\tau_{L}}{\pi \cdot \alpha }}\left(T_{vap}-T_{0}\right)\tan\theta }{\rho \cdot \left[C_{pl}\cdot \left(T_{vap}-T_{0}\right)+h_{lv}\right]},$$

where *l*_max_ is the obtained maximum depth of the hole, which can be calculated by the given thermophysical parameters of material and laser processing parameters. For different pulse width, the measured and theoretical hole depth are shown in Figure 15.

It can be seen that the experimentally measured hole depth was much larger than that by calculation when pulse width was less than 2 ms. This is because, in Equation (10), energy absorption by the ejected debris is ignored during the keyhole evolution. The depth increment of the hole only by vaporization is much smaller. Vaporization only contributes to 20–30% of the depth increment of the hole. Conversely, ejected debris contributes to over 60% of the depth increment of the hole. It is proven that ejection is the main material removal mechanism for the rapid increment stage during the first 2 ms. When the pulse width exceeded 2 ms, it can be noticed that the experimental hole depth was closer to that by calculation. Vaporization contributed to about 80% depth increment of the hole in this stage, indicating that evaporation was dominant for the material removal during the slow increment stage.

From above discussion combining the dimensionless laser power density and comparison between the experimental and theoretical maximum hole depth, it can be concluded that, during the first 2 ms, there is a rapid increment of the keyhole size. At this stage, the material is mainly removed by the ejected debris. When the pulse width exceeds 2 ms, there is a slow increment of the keyhole size. Vaporization is the main material-removal way at this stage.

For single-pulse laser drilling on PEEK, the effect of processing parameters on the morphology of micro-holes was analyzed in detail, which not only provides a processing window for laser drilling of PEEK but also gives a guide for the drilling process using other polymers (PET, PI, PC, PS, PMMA). The dynamic process of keyhole evolution was observed during the laser–PEEK interaction. Ejection and vaporization were proposed as the main material removal mechanisms. It is useful to understand the physical processes of laser drilling on polymers with a long pulse and infrared wavelength. Moreover, it provides a theoretical basis to optimize processing parameters, in order to achieve micro-holes with small diameter, high aspect ratio, and large material removal rate in aerospace and biomedical applications.

## 4. Conclusions

For single-pulse laser drilling on PEEK, a series of experiments were conducted to study the effect of pulse width and peak power on the geometrical morphology of the hole, which mainly focused on the hole depth, hole diameter, and aspect ratio. Moreover, the keyhole evolution was observed by a time-resolved system to understand the physical processes. The material removal mechanism of laser–PEEK interaction was proposed. Some main conclusions can be summarized as follows:

- A method for observing morphologic characteristics of the micro-hole was proposed and applied successfully, especially in the field of laser drilling. The hole depth and hole diameter could be measured rapidly right after drilling experiments on two PEEK samples jointed together with a “sandwich” structure clamp.
- Across all the processing parameters, the maximum drilling depth was 7.06 mm. The hole diameter varied from 200 μm to 400 μm. The maximum aspect ratio of the hole was 23 when the depth was 7.06 mm and diameter was 0.31 mm. During the first 2 ms, the hole depth increased rapidly with the increase in pulse width at a rate of 2.1 m/s. When the pulse width ranged from 2 ms to 4 ms, a slow increment rate around 0.3 m/s was obtained. After 4 ms, the hole depth was almost unchanged.
- The keyhole evolution process could be divided into three stages: rapid increment stage (0–2 ms), slow increment stage (2 ms–4 ms), and stable stage (>4 ms). Keyhole evolution was dominated by ejection during the rapid increment stage, while it was dominated by vaporization during the slow increment stage.
- The laser–PEEK interaction was mainly influenced by the photothermal effect. The dimensionless laser power density is defined as the ratio of laser absorption to the vaporization used for the material removal. Ejected debris contributed to over 60% of the depth increment of the hole at the rapid increment stage, whereas vaporization contributed to about 80% of the depth increment of the hole at the slow increment stage. It can be concluded that the material removal mechanism was dominated by ejection and vaporization during the rapid and slow increment stages, respectively.

## Figures and Tables

**Table 1:** Thermophysical properties of PEEK [22,33].

Thermophysical Properties	Value
Density	1.3 × 10^3^ kg/m^3^
Glass transition temperature (T_g_)	416 K
Melting point	616 K
Vaporization temperature	623 K–773 K
Coefficient of thermal expansion	4.7 × 10^−5^/K
Specific heat	2.2 kJ/(kg·K)
Diffusivity	1 × 10^−4^ m^2^/s
Conductivity	0.29 W/(m·K)
Absorption coefficient	0.68
